# Tumor Temporal Proteome Profiling Reveals the Immunological Triple Offensive Induced by Synthetic Anti-Cancer *Salmonella*

**DOI:** 10.3389/fimmu.2021.712936

**Published:** 2021-08-19

**Authors:** Shuxin Yang, Wenjuan Zhao, Muchun Zhu, Huijuan Hu, Weijie Wang, Zhongsheng Zang, Meiling Jin, Jiacheng Bi, Jiandong Huang, Chenli Liu, Xuefei Li, Peng Yin, Nan Li

**Affiliations:** ^1^Chinese Academy of Sciences (CAS) Key Laboratory for Quantitative Engineering Biology, Shenzhen Institute of Synthetic Biology, Shenzhen Institute of Advanced Technology, Chinese Academy of Sciences, Shenzhen, China; ^2^Guangdong-Hong Kong-Macao Joint Laboratory of Human-Machine Intelligence-Synergy Systems, Shenzhen Institute of Advanced Technology, Chinese Academy of Sciences, Shenzhen, China

**Keywords:** quantitative proteomics, cancer immunotherapy, engineered *Salmonella*, blood coagulation, phagocytosis, antitumor T cell response

## Abstract

The engineered “obligate” anaerobic *Salmonella typhimurium* strain YB1 shows a prominent ability to repress tumor growth and metastasis, which has great potential as a novel cancer immunotherapy. However, the antitumor mechanism of YB1 remains unelucidated. To resolve the proteome dynamics induced by the engineered bacteria, we applied tumor temporal proteome profiling on murine bladder tumors after intravenous injection of either YB1 or PBS as a negative control. Our data suggests that during the two weeks treatment of YB1 injections, the cured tumors experienced three distinct phases of the immune response. Two days after injection, the innate immune response was activated, particularly the complement and blood coagulation pathways. In the meantime, the phagocytosis was initiated. The professional phagocytes such as macrophages and neutrophils were recruited, especially the infiltration of iNOS^+^ and CD68^+^ cells was enhanced. Seven days after injection, substantial amount of T cells was observed at the invasion margin of the tumor. As a result, the tumor shrunk significantly. Overall, the temporal proteome profiling can systematically reveal the YB1 induced immune responses in tumor, showing great promise for elucidating the mechanism of bacteria-mediated cancer immunotherapy.

## Introduction

Globally cancer is an important leading cause of death, accounting for nearly 10 million deaths in 2020 alone (WHO). The vast majority of cancers are solid tumors, which develop in a variety of organs, such as breast, lung, colorectum, liver, prostate and bladder etc. To date, the conventional methods of cancer treatment such as surgery, radiation therapy and chemotherapy are still the preferred choice. However, surgery is not an effective therapy for metastatic cancer and needs to be used in combination with other traditional therapies such as radiation or chemotherapy. The procedure and therapeutic effects of radiotherapy and chemotherapy are impeded by the necrotic and hypoxic regions in tumors (1). Cancer immunotherapy had emerged as a standard treatment with great potential in cancer therapy. It includes adoptive T cell therapy, immune checkpoint inhibitors and cancer vaccines etc. The earliest cancer immunotherapy could be traced back to the bacteria-mediated cancer therapy 200 years ago (2). So far, various kinds of bacterial infection or injection have been reported to relieve cancer symptoms or were specifically used for cancer treatment, such as *Streptococcus pyogenes* (*S. pyogenes*), Coley’s toxins, *Clostridium histolyticum* and the Bacillus Calmette-Guerin (BCG) vaccine (3–7).

Recent progress in the fields of immunology and biotechnology has generated new interest in the modification of tumor-targeting bacteria, returning them to the forefront of cancer research (8). Many non-pathogenic obligate anaerobes and facultative anaerobes have been shown selectively proliferate in tumor cells possessing hypoxia and abnormal angiogenesis. Attenuated *Salmonella* is an outstanding example of one such obligate anaerobe (9–12). In our previous studies, synthetic biology techniques were applied to construct an engineered “obligate” anaerobic *Salmonella typhimurium* strain YB1 (*Salmonella* YB1). The engineered *Salmonella* YB1 acted as an obligate anaerobe, targeting the hypoxic and necrotic regions in tumors and significantly suppressing the growth and metastasis of a broad range of cancers, including bladder tumor (13), neuroblastoma, liver cancer and breast cancer (14–17). Systemic administration of *Salmonella* YB1 can effectively stimulate the immune system, resulting in the increased production of tumor necrosis factor-α (TNF-α), and interferon-γ (IFN-γ), as well as activation of both innate and adaptive immune cells (14–17). These stimulated immune responses might create a hostile environment for tumor progression (18). However, the underlying systemic therapeutic mechanism of *Salmonella* YB1 remains to be elucidated.

The proteomic approach is a promising technique that can facilitate the systematic characterization of the proteome dynamics in pharmacology and interspecies interactions (19–21). Systematically revealing the dynamics of the tumor proteome after YB1 treatment will contribute to understanding its mechanism. The quantitative proteomics of tumors undergoing bacterial treatment can especially contribute to understanding of the interaction process between bacteria and tumors. We profiled the temporal proteome of the bladder tumor xenografts on mice after *Salmonella* YB1 injection. We then applied a label-free quantitative proteomic approach with in-solution digestion to identify the differentially expressed proteins by mass spectrometry analysis. The tumors with YB1 injection experienced three distinct phases of immune responses, including the activation of complement and blood coagulation pathways, iNOS^+^ and CD68^+^ cells mediated phagocytosis, and the accumulation of T cells at the invasion margin of tumors. In summary, we performed the systematic temporal analysis to the proteome of the tumor with YB1 treatment. It will be beneficial to reveal the therapeutic mechanism of YB1 and its application in cancer immunotherapy in the future.

## Materials and Methods

### *Salmonella* YB1 and Tumor Cells Culture

*S. typhimurium* strain YB1 was cultivated in Luria-Bertani (LB) broth containing 25μg/mL chloramphenicol at 37°C, with shaking at 220 rpm overnight. The YB1 cultures were then transferred twice and grown until the logarithmic phase. OD600 was measured to determine the bacterial count. The MB49 mouse bladder cancer cell line was maintained in Dulbecco’s modified Eagle’s medium-high glucose (DMEM-HG) supplemented with 10% FBS (fetal bovine serum), 1% streptomycin and 1% penicillin. The medium was renewed every other day. Cells were cultivated at 37°C in a humidified atmosphere of 5% CO_2_.

### Animals and Tumor Tissues Collection

All animal experiments were approved by the animal care regulations of the Institutional Animal Care and Use Committee of the Shenzhen Institutes of Advanced Technology, Chinese Academy of Sciences. Four-to six-week-old female C57BL/6 mice (Vital River Laboratory Animal Technology Co. Ltd, CHN) were subcutaneously injected with MB49 cells (1×10 ^6^) in the flank region. Tumor volume was calculated according to the following formula: tumor volume = length × (width) 2/2. When the average volumes of the MB49 tumors reached approximately 200 mm^3^, the C57BL/6 mice were randomly divided into two groups. One group was inoculated *via* the tail vein of the mice with YB1 (1×10 ^7^) dissolved in 125μL PBS, whereas the control group was treated with 125μL PBS only. The mice were killed at different time points after injection. The whole tumor tissues were washed twice with ice-cold phosphate-buffered saline (PBS) to remove blood and other contaminants, quick-frozen in liquid N_2_ and stored at –80°C for protein extractions.

### Protein Preparation and Peptide Extraction

At least 1 mg samples of tumor tissues were cut off and lysed in a buffer that consisted of 5mM EDTA, 150mM NaCl, 20mM HEPES (pH8.5), 1% SDS, and a Roche complete protease inhibitor cocktail tablet for 10 min on ice. Following lysis, tissues debris was further lysed using sonication under suitable conditions. The lysates were centrifuged at 20,000 g for 15 min at 4°C, and the supernatants were harvested. The protein concentration was measured by BCA Assay Kit (Thermo Fisher Scientific, P/N 23225). Approximately 100μg protein solution was reduced and alkylated for 30 min at 37°C using 1μl 0.5 M TCEP, and 2μl 1 M CAA. After alkylation, quantitative precipitation of soluble and hydrophobic proteins from dilute solutions was based on a defined methanol-chloroform-water mixture method as described in Wessel D et al. (22). In brief, an aliquot (0.200μl) of methanol was added to 50μl of protein sample (approximately 100μg proteins) and the samples were vortexed. Then, chloroform (50μl) was added and the samples were vortexed again. For phase separation, 150μl of water (HPLC grade) was added, and the samples were vortexed vigorously and centrifuged at 9000 g for 2 min at room temperature. The upper phase was carefully removed and discarded. A further 150μl methanol was added slowly to the rest of the lower chloroform phase and the interphase with the precipitated protein. The samples were mixed gently and centrifuged again at 9000 g for 2 min at room temperature to pellet the protein. The supernatant was removed and the protein pellet was dried under a stream of air for 5 min. The protein pellets were re-disolved by the addition of 20μl of Urea buffer (8 M Urea, 100mM HEPES) and vortexed fully. The protein pellets were reconstituted by the addition of 180μl of 20mM HEPES and vortexed fully. For digestion, 2μg mass spectrometry grade trypsin (Promega, P/N V5280) were added for digestion overnight at 37°C. The peptide digestions were quenched by 10μl of 10% formic acid (FA).

### Peptides Desalting

The acidifying peptide samples were desalted using a 100 mg desalting column (Thermo Fisher Scientific, P/N 60108-302). In short, the desalting column was activated with 1 ml acetonitrile (ACN)) twice, and 1 ml buffer B (80% ACN, 0.5% FA in water) twice. The buffer A was loaded twice to equilibrate the desalting column. Subsequently, the peptide solution was loaded onto the column, washed with 1 ml buffer A three times and eluted with buffer B to a clean tube. The eluent was dried completely in a SpeedVac centrifuge at 45°C and store at -80°C.

### LC-MS/MS Analysis

All peptides were reconstituted in 0.1% FA (vol/vol) and separated on on reversed-phase columns (trapping column: particle size = 3 μm, C18, length = 20 mm (Thermo Fisher Scientific, P/N 164535); analytical column: particle size = 2 μm, C18, length =150 mm(Thermo Fisher Scientific, P/N 164534)) on an Ultimate™ 3000 RSLCnano system (Thermo Fisher Scientific, San Jose, CA, USA) coupled to Orbitrap Q-Exactive™ HF (Thermo Fisher Scientific). Peptide separation was achieved using a 120 min gradient (buffer A: 0.1% FA in water, buffer B: 0.1% FA in 80% ACN) at a flow rate of 300 nl/min, then analyzed by Orbitrap Q-Exactive™ HF in a data-dependent mode. The Orbitrap Q-Exactive™ HF mass spectrometer was operated in positive ion mode with the ion transfer tube temperature set at 320°C. The positive ion spray voltage was 2.1 kV. Full-scan MS spectra (m/z 350–2000) was acquired in the Orbitrap with a resolution of 60,000. HCD fragmentation was performed at normalized collision energy of 28%. The MS2 automatic gain control (AGC) target was set to 5e4 with a maximum injection time (MIT) of 50 ms and dynamic exclusion was set to 45 s.

### Proteomics Data Processing and Bioinformatic Analysis

The MS/MS data were searched against a Swiss-Prot database (Musculus release-20190412 and Salmonella release-20190426 downloaded from UniProt) with MaxQuant 1.5.3.30. Data were searched with a precursor mass tolerance of 20 ppm and a fragment mass tolerance of 0.5 Da. Searches were performed with enzyme specificity and only tryptic peptides were allowed to remain in the final data sets, and up to two mis-cleavages allowed. Cysteine carboxamidomethylation was specified as a static modification; oxidation of methionine residue and acetylation, (protein-N) were allowed as variable modifications. Reverse decoy databases were included for all searches to estimate false discovery rates. Peptide and protein identifications were also quantified and filtered for less than 1% false-discovery rate (FDR).

The intensity values from MaxQuant were normalized and further processed using the VSN method. We removed proteins with fewer than two samples in each group of samples at each time point. Then, the missing values were imputed by using the QRILC method. The Limma package was used for determining differentially expressed proteins between tumor mice and tumor mice injected with Salmonella. Proteins with an average fold change >1 and *p*-value <0.05 were considered different. For proteins of significant difference, their molecular functions and associated biological processes were analyzed using the DAVID (https://david.ncifcrf.gov/home.jsp) analysis tool, the FDR threshold was set at 5%. In order to observe the protein change panels at seven time points, we used the CLUSTER package (23) to create a *k*-means cluster analysis.

### Immunohistochemical Analysis

Tumor tissues were collected from MB49-bearing mice intravenously injected with bacteria (1x10^7^ CFU per mice) or PBS for hematoxylin and eosin (H&E) staining. Macrophages were labeled with F-4/80 antibody (Servicebio, GB11027). M1 subset macrophages were marked with iNOS and CD68 antibody (Servicebio, GB11119, GB11067). Neutrophils were labeled with Ly-6G antibody (Servicebio, GB11229). The complement activity was recognized by C3 antibody (Abcam, ab200999). T and B cells were labeled respectively with CD3 antibody (Servicebio, GB13014) and CD19 antibody (Servicebio, GB11061). The collagen was stained with Sirius red staining. The mouse primary antibodies was detected using a goat anti-mouse secondary antibody (Servicebio, gb111739).

### Quantification of the Immunohistochemical Staining

Expression levels of effector proteins as well as the abundance of various type of immune cells were quantified using images of tumor sections with immunohistochemical staining. Nine regions of interest (ROI) were manually selected from the whole scan image of the each tumor under 40x magnification (about 250um x 480 um), and there are three tumor-bearing mice for each experimental condition. First, we adopted a color deconvolution model (24) to separate the unstained and stained regions into separate channels with the deconvolution matrix set as [0.650 0.704 0.286; 0.268 0.570 0.776; 0.711 0.423 0.561]. Then, image binarization with an appropriate threshold setting (C3 w/70, CD3 w/10, CD11c w/30, CD68 w/20, F4/80 w/70, iNOS w/70, Ly6G w/25 and CD19 w/10) were applied to extract the area of different markers. Next, nuclei boundaries were detected by using a level set segmentation and detection technique as previously described (25). Note that we used the area of cell nucleus instead of cytoplasm to represent the area of cell regions, for it is more difficult to define the regions of cytoplasm, and we hypothesized that the nucleus/cytoplasm area-ratio is consistent across different regions. Finally, the area of stained signals (S) and all cell nucleus (N) can be obtained, and a ratio of S to N (S/N) was defined to evaluate the expression levels of effector proteins or the abundance of various type of immune cells in the tumor-section images.

### T Cell Separation and ELISpot Assay

The mouse spleen was separated and grinded in RPMI1640 medium, then the T cells were isolated by magnetic negative selection using the CD8^+^ T cell isolation kit (Miltenyi Biotec.). The activity of CD8^+^ T cells was tested using the Mouse IFN-γ precoated ELISPOT Kit (DAKEWE, 2210005) as the described in the product manual.

1×10^5^ freshly spleen CD8^+^ T cells were plated in triplicates into 96-well Elispot plates precoated with anti-mouse-IFN-γ antibody and stimulated for 18 h at 37°C under 5% CO_2_ with either 1×10^4^ MB49 cells or 1×10^5^ YB1 resuspended in PBS (positive control). As unstimulated control, cells were incubated for 18 h in cell culture medium (background value). The PMA stimulation was used as a positive control as well. After cell removal, plates were incubated with biotinylated antibody and streptavidin-HRP respectively for 1 h. Spot detection was performed by FluoroSpot and ELISpot Reader (Mabtech IRIS™).

### Statistical Analysis

All values are expressed as means ± SD. Statistically significant differences among individual treatments and the corresponding control groups were determined by the Kolmogorov–Smirnov test (K–S test or KS test) or analysis of variance (ANOVA). Experiments were independently repeated at least three times. All analyses were carried out using GraphPad Prism 9. A *p*-value <0.05 was considered to be statistically significant.

## Results

### Temporal Proteome Profiling Unraveling Diverse Tumor Responses During YB1 Treatment

**Figure 1:**
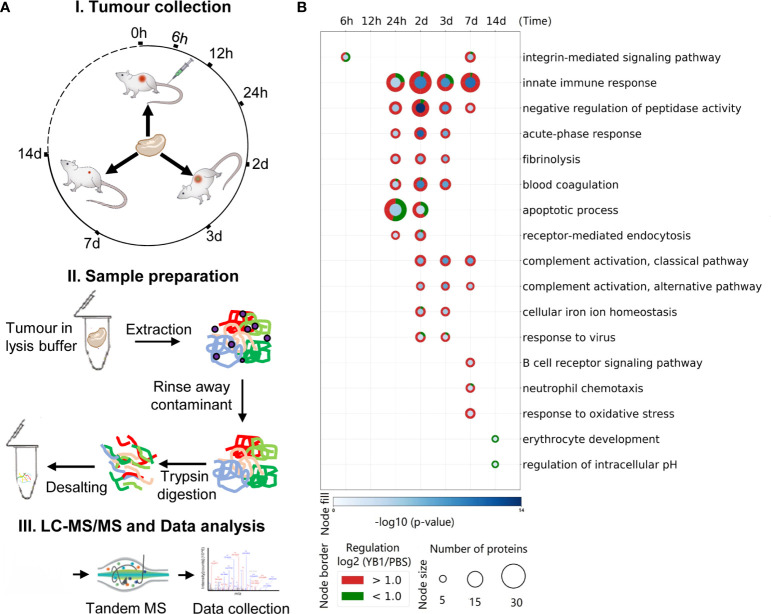
Temporal proteome profiling for exploring the anti-cancer mechanism of *Salmonella* YB1. **(A)** Experimental design and data analysis workflow of the tumor proteome. (I) Illustration of the tumor collection after *Salmonella* YB1 strain treatment at different time points. (II). General workflow of proteomic sample preparation process. (III) The workflow of MS-based quantitative proteomics and bioinformatics analysis. **(B)** GO term enrichment of differentially expressed tumor proteins. Selected enriched GO terms are depicted; all enriched GO terms can be found in **Supplementary Table 1**. Node size and colors depict significance, [–log10 (*p*-value)] right-sided hypergeometric test, Bonferroni corrected) (blue shading) and number of proteins (Node size), respectively. n = 4 biologically independent samples.

To build the tumor model, C57BL/6 mice were subcutaneously injected with MB49 cells in the flank region (detail in method). In brief, when the average size of the MB49 tumors reached approximately 200mm^3^, the C57BL/6 mice were randomly divided into two groups and injected intravenously with *Salmonella* strain YB1 (treatment group) or PBS (control group). The tumor growth was inhibited by YB1 treatment (**Supplementary Figure 1**), same as our previous study (13). To map the proteomic atlas of the *Salmonella* YB1 treatment bladder tumors, we collected the whole tumors in four replicates from each group at each time point. There were seven time points in total, including 6 hours, 12 hours, 24 hours, 2days, 3 days, 7 days and 14 days post *Salmonella* YB1 or PBS injection. We applied a label-free quantitative proteomic approach with in-solution digestion to 56 identified samples by mass spectrometry analysis (**Figure 1A**). To better understand the molecular functions of tumor proteins during *Salmonella* YB1 treatment, GO term enrichment analysis for YB1 proteins displaying ≥1 ± log2(fold-change) revealed a total of 454 enriched GO terms (*p ≤* 0.05, right-sided hypergeometric test, Bonferroni corrected), with 397 being upregulated and 57 downregulated (**Figure 1B**, **Supplementary Table 1**).

We observed a number of infection-related GO terms were significantly enriched, as well as the integrin-mediated signaling pathway related to bacterial invasion at 6 hours (20), an apoptotic pathway at 24 hours and response to virus at 2 days. These findings demonstrate that YB1 could invade the tumor and cause cell apoptosis, consistent with our previous study (14). Moreover, a strong innate immune response occurred at 24 hours last to 7 days, including acute-phase response, blood cogulation and complement activation etc. (**Figure 1B**, **Supplementary Table 1**). Overall, our results are in line with previous observation in Salmonella based tumor therapy, where Salmonella demonstrates an intrinsic anti-tumor effect, largely attributed to its immune stimulatory activity, as well as activation of both innate and adaptive immune cells (18, 26).

### A Dynamic Proteomic Atlas of the YB1 Treated Tumors

**Figure 2:**
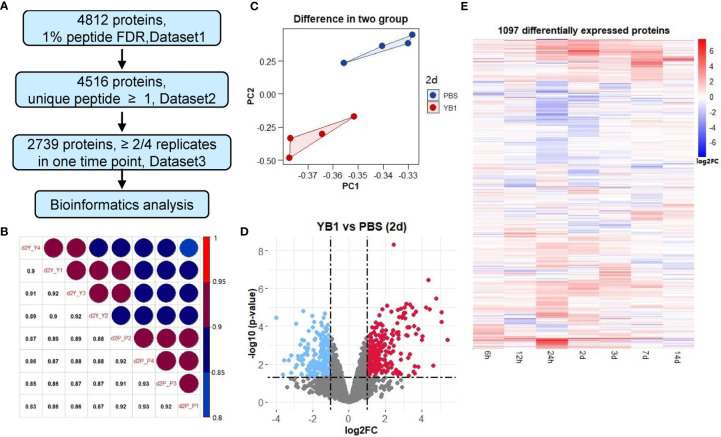
Quantitative proteomics revealed differential protein expressions in tumor induced by YB1 treatment. **(A)** Multiple data sets with different filtering criteria. **(B)** Replicate correlation of YB1 *versus* PBS samples for the indicated time points (2 day). **(C)** Principal component analysis of the proteomics data at 2 day. Each dot represents an independent biological replicate. **(D)** Analysis of significantly dysregulated proteins in YB1 (determined by *t*-test). Volcano plots of YB1 *versus* control comparisons. *p-*values (-log base 10) are plotted as a function of the proteins ratio (log base 2) for YB1 *versus* PBS group. **(E)** Hierarchical clustering of differentially expressed proteins by semi-supervised, Ward’s hierarchical clustering.

Summing up all the 56 samples, 4,812 proteins were quantified at a 1% peptide FDR (false discovery rate) (**Figure 2A**, **Dataset1** in **Supplementary Table 2**). A total of 4,516 proteins were identified as high-quality IDs by selecting those that have been measured with at least one unique peptide (**Figure 2A**, **Dataset2** in **Supplementary Table 3**). Further filtering for proteins identified in at least 2 of the 4 replicates at one time point resulted in a final list of 2,739 proteins for bioinformatic analyses (**Figure 2A**, **Dataset3** in **Supplementary Table 4**). Replicate correlation of samples showed good consistency and a high degree of correlation (R = 0.85–0.93) between the YB1 and PBS experiments (**Figure 2B**, **Supplementary Figure 2**). Principal component analysis (PCA) showed that replicates clustered closely and the YB1 separation from control clusters PBS (**Figure 2C**, **Supplementary Figure 3**). Overall, our tumor proteomic dataset is of high quality and reproducibility.

After these filtering steps, we performed relative protein quantification based on the log2 fold change of protein intensity of YB1 versus PBS samples and *p*-value from the Limma test (**Figure 2D**, **Supplementary Figure 4**). This analysis showed that, 1097 out of the 2739 quantified proteins were significantly up or down regulated leastwise at one timepoint (*p*-value ≤0.05, |log2 fold change| ≥1) (**Supplementary Table 5**). These results revealed that the majority of differential expression of proteins occurred from 24 hours to 7 days (947 of 1097). Most notably, at 24 hours and 2 days, there were 453 and 412 differentially expressed proteins respectively (**Figure 2E**).

### Key Immune Responses Revealed by K-Means Clustering Analysis

**Figure 3:**
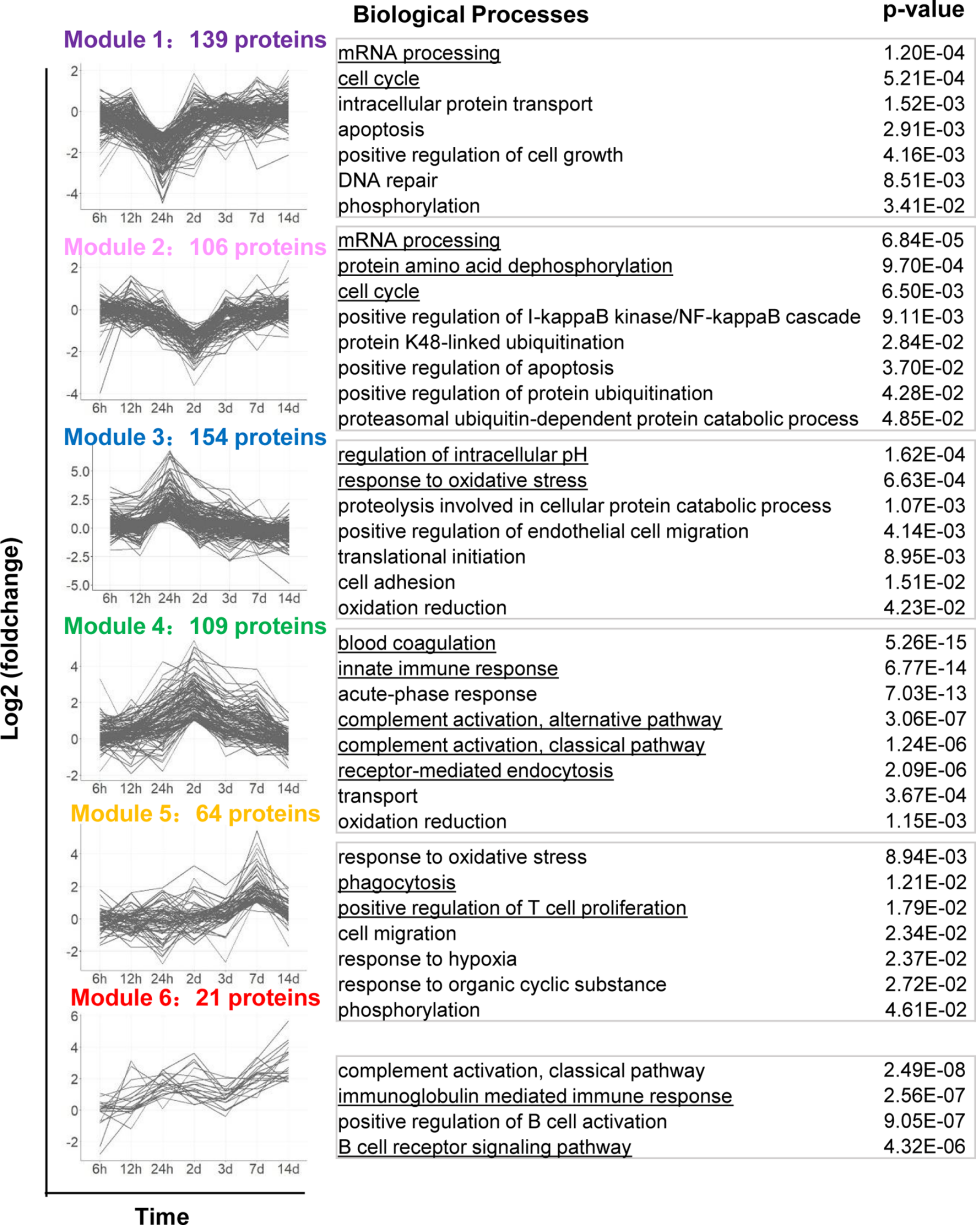
Dynamic protein co-expression modules classified by *k*-means analysis with GO annotation. The left panel shows the six modules classified by the *k*-means clustering analysis, according to their dynamic co-expression patterns. x-axis represents sampling time, y-axis represents log2 fold-change of protein intensity. The right panel shows the corresponding GO Biological Process terms to each module.

To better exhibit biological processes induced by YB1 treatment, we applied the *k*-means algorithm to classify the 1097 differentially expressed tumor proteins, according to their dynamic co-expression patterns. We obtained six protein modules with the unsupervised k-mean clustering analysis (**Supplementary Table 6**). Then we analyze GO Biological Process terms in each of the modules (**Figure 3**, **Supplementary Table 7**).

Proteins in Module 1 and 2 were gradually downregulated, dropped to a trough at 24 hours and 2 days respectively, including proteins of RAD50, CCAR2, CDK5 etc. These proteins are mainly involved in cell-cycle, including DNA repair and regulation of cell grows. It has recently been shown that cell-cycle are correlated with *Salmonella* intracellular proliferation (20). The suppression of cell-cycle may indicate a host-driven response to *Salmonella*. Module 2 proteins also participate in the post-translational modification of proteins, such as dephosphorylation and ubiquitination. It appears that cell proliferation and protein degradation are the predominant downregulated pathways during *Salmonella* YB1 treatment.

Proteins in Module 3, 4, 5 and 6 were progressive upregulated and respectively peaked at 24 hours, 2 days, 7 days and 14 days. Module 3 proteins were mainly involved in response to oxidative stress, endothelial cell migration and regulation of intracellular pH. The latter behavior is consistent with the previous study that protein synthesis and cell proliferation and mitosis could make a change in cytosolic pH (27). Although proteins in Module 4-6 peaked at different time point, they were significantly enriched in similar biological processes including innate and adaptive immune response. In particular, we detected a strong response to blood coagulation and complement activation (C3, C9, C4, C5, etc.) in Module 4. This finding indicated that YB1 treatment would trigger blood coagulation within the tumor, consistent with the previous report (23).

We also observed strong induction of endocytosis such as phagocytosis signaling response in both Module 4 and 5, including proteins iNOS, CD14, CD177, NGP, CD68, etc. CD14, which is well known mediate the innate immune response to bacterial lipopolysaccharide (28), was gradually upregulated from 12 hours, peaked at 2 days (+2.91 Log2 Fold change, adjusted *p*=3.38E-05). The expression of iNOS reached a peak at 2 days (+2.84 Log2 Fold change, adjusted *p*=2.75E-03). As a marker of M1 type macrophages, iNOS produces nitric oxide (NO) which mediates tumoricidal and bactericidal activity (29). Likewise, CD177 and NGP was upregulated from 24 hours and peaked at 2 days. CD177 plays an important role in neutrophils recruitment caused by bacterial infection *in vivo* (30). NGP plays a role as a negative regulator of tumor vascular development and metastasis (31) (**Figure 1B**). Proteins in Module 5 were also enriched in positive regulation of T cell proliferation (32). Proteins in Module 6 were involved in B cell receptor signaling pathway and immunoglobulin mediated immune response. Together, these findings highlighted the roles of *Salmonella*-induced immune responses in bacteria mediated anti-tumor effects. However, the functional significance of many of these proteins will require further study.

### Thrombosis and Complement Were Initiated After YB1 Treatment

**Figure 4:**
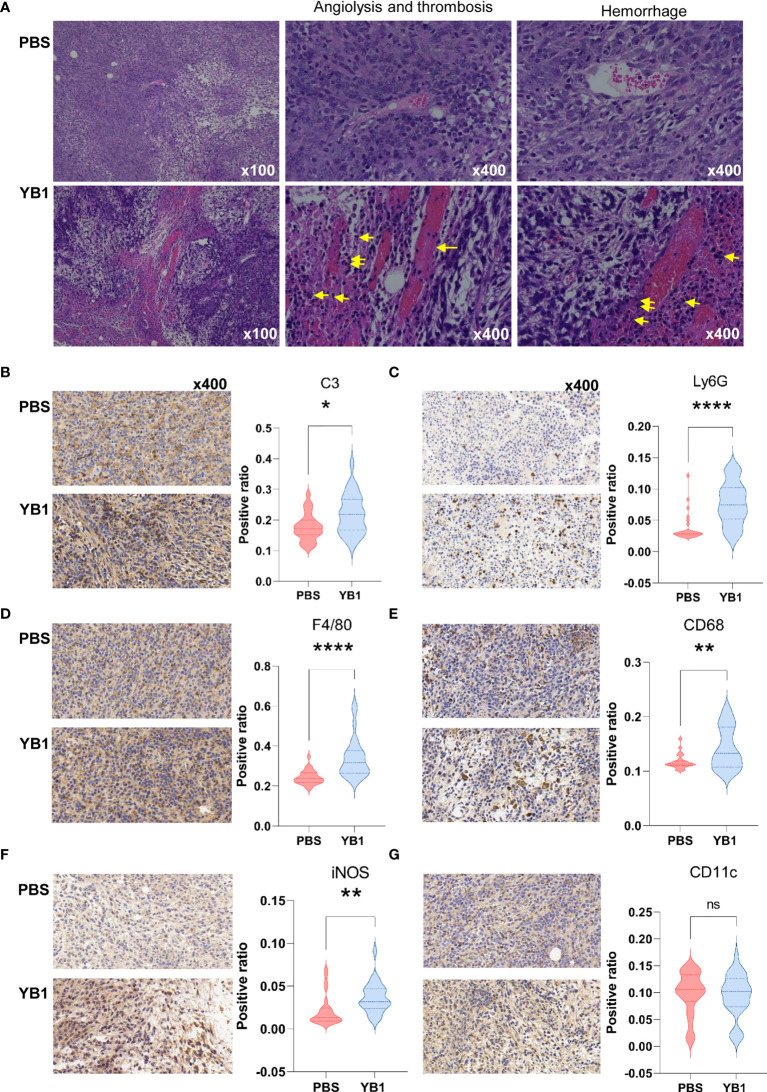
*Salmonella* YB1 induced the innate immune responses in tumors. **(A)** Representative images of hematoxylin and eosin (H&E)–stained tumor slices collected from mice injected with phosphate-buffered saline (PBS) or YB1 at 2 days post injection. The yellow arrow indicates angiolysis, thrombosis and neutrophils infiltration. Area ratio (see methods) of **(B)** C3^+^ cells, **(C)** Ly6G^+^ cells, **(D)** F4/80+ cells, **(E)** CD68^+^ cells, **(F)** iNOS^+^ cells, **(G)** CD11c^+^ cells based on immunohistochemical-stained images. Three biological replicates of images with nine random views under 40x magnification per image were collected for statistical analysis which was performed using the K–S test.*p < 0.05, **p < 0.01, ****p < 0.0001, ns, no significant difference.

To confirm the complement and blood coagulation in the proteomics analysis results, we performed the immunohistochemical analysis to the mice tumor tissues from the YB1 and PBS injection groups. We collected the tumors of the mice injected with YB1 and PBS at 2 days post injection for hematoxylin and eosin (H&E) staining (**Figure 4A**). In a large view, we could find severe hemorrhagic inflammation in the tumor on mice injected with YB1. Compared to the control group, the mice with YB1 injection showed significant erythrocyte diapedesis in tumors, along with inflammatory cell infiltration. Erythrocyte could be abundantly found inside the tumor after bacterial infection (**Figure 4A**). The results above are representative features in bacterial infection. We observed that the colonization of bacteria in tumor could trigger thrombosis by disrupting tumor blood vessels, which was consistent with the previous study (33). On the other hand, we performed the immunohistochemical analysis to C3, a canonical marker of complement, in the tumor slices. It showed that the expression of C3 in tumor with YB1 injection is significantly higher than the control group (**Figure 4B**). As the description above, not limited to C3, a series of complement related proteins, such as Serpind1, Cfd, C5, C1qbp, etc., were upregulated in tumor with YB1 treatment. The immunohistochemical results confirmed the proteomics analysis and showed that YB1 injection could initiate the activation of complement and blood coagulation in tumor. The activation of the complement system could also induce the recruitment of macrophages and neutrophils into tumor tissues (34, 35), which may indicate a tumoricidal activity.

### Phagocytosis Were Activated After YB1 Treatment

We performed immunohistochemical analysis to the professional phagocytes in the tumors at 2 days post injection. F4/80 and CD68 markers were used to identify macrophages. F4/80 is well-known as a highly restricted macrophage molecule in mice. CD68 is exploited as a valuable cytochemical marker to immunostain monocyte/macrophages in the histochemical analysis of tumor tissues. The results showed that there were significantly higher numbers of F4/80^+^ cells and CD68^+^ cells in tumors with YB1 injection than the control group (**Figures 4D, E**). In addition, the results showed there was an increased number of the iNOS^+^ cells in the immunohistochemical slices of YB1 injected tumors (**Figure 4F**). This result consisted with the observation in the above proteomics data, where the iNOS was upregulated at 2 days post YB1 injection (**Figure 4F**, **Supplementary Table 5**). These results further confirmed our proteomic analysis results.

Then we detected another two typical phagocytes, neutrophils and dendritic cells (DCs). We detected the expression of Ly6G and CD11c in the tumor slices. Ly6G is a marker for monocytes, granulocytes and neutrophils. CD11c is the most widely used defining marker for dendritic cells (DCs). The results showed that there was more Ly6G^+^ cells in tumors with YB1 injection than the control group. But there was no significant difference of the CD11c^+^ cell number in these two groups (**Figures 4C, G**). These results indicate that the YB1 could induce neutrophils and macrophages gathering, which mediated phagocytosis in tumors. In addition, iNOS is often used as a marker of M1-like macrophages (36), its upregulation may indicate the possible enrichment of M1 macrophages.

### T Cells Accumulated at the Invasion Margin of the Tumor After YB1 Treatment

**Figure 5:**
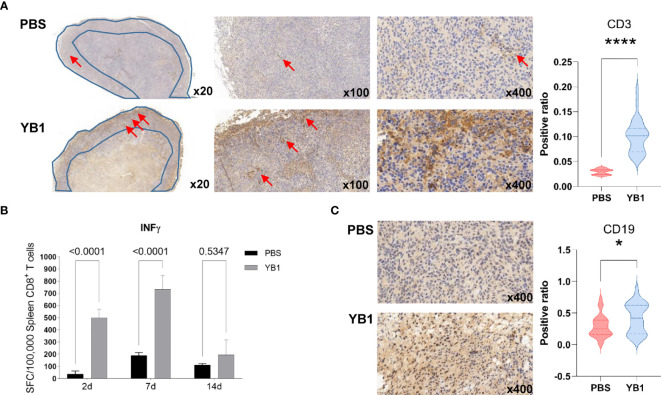
*Salmonella* YB1 induced the adaptive immune responses in tumors. **(A)** Representative images of immunohistochemical stained tumor slices collected from mice injected with phosphate-buffered saline (PBS) or YB1 at 7 days post injection. The area of the blue polygon indicates the invasive margin of the tumor, the red arrow represents CD3^+^ T cells. An S/N ratio of CD3^+^ cells in the invasion margin of tumors. **(B)** CD8^+^ T cells from YB1 (gray) and PBS (black) treated mice spleen were stimulated by MB49 cells. The background value of IFN-γ expression of CD8^+^ T cells has been removed. IFN-γ ELISpot results of three replicates are expressed as mean SFC (IFN-γ-specific spot-forming cells)/100,000 CD8^+^ T cells. Error bars represent standard deviations. Statistical analysis was performed using the two-way ANOVA with Šidák’s multiple comparisons test. **(C)** A ratio of CD19^+^ cells versus total cell numbers in the tumors. Statistical analysis in **(A, C)** was performed using the K–S test. *p < 0.05, ****p < 0.0001.

In our proteomics data, the positive regulation of T cell proliferation was significantly upregulated at 7days post YB1 injection. To further confirm this result, we performed the immunohistochemical staining to the CD3 of the T cells in the mice tumor sections. The CD3 protein complex is a defining feature of T cell lineage. Compared to the tumors in the control group, we found that the T cells in the tumors with YB1 injection accumulated at the invasion margin of the tumors, and the CD3^+^ T cells in the tumors with PBS injection tend to congregate at the tumor center. And the number of T cells at the margin area in YB1 injection group was significantly higher than that in the control group (**Figure 5A**). As previously reported, the location of T cells, such as at the invasive margin, rather than the center of the tumor, is an important positive predictor of outcome (37, 38). These results may indicate a good prognosis of the tumors with YB1 injection. Moreover, we compared the antitumor effects of CD8^+^ T cells in the spleens of the YB1 treated and control mice. The CD8^+^ T cells were isolated from mice spleen and stimulated with MB49 mouse bladder tumor cells for 18 hours. IFN-γ secreted by spleen CD8^+^ T cells was measured through IFN-γ ELISpot assay. We found that the IFN-γ secretion by the spleen CD8^+^ T cells in the YB1 treated mice was significantly higher than that in the control group (**Figure 5B**). These results indicate YB1 could promote the antitumor T cell effects in the tumor-bearing mice.

### B Cells Accumulated in the Tumor After YB1 Treatment

At two weeks after YB1 treatment, the B cells related signaling pathways were activated in the proteomics profiling. We detected the expression of CD19, a biomarker for normal and neoplastic B cells, in YB1 injected mice tumors at 14 days. The results showed that there were more CD19^+^ B cells in the YB1 injected mice tumor than the control group (**Figure 5C**). These results demonstrate that *Salmonella* YB1 strain could induce the recruitment of B cells in tumors.

## Discussion

**Figure 6:**
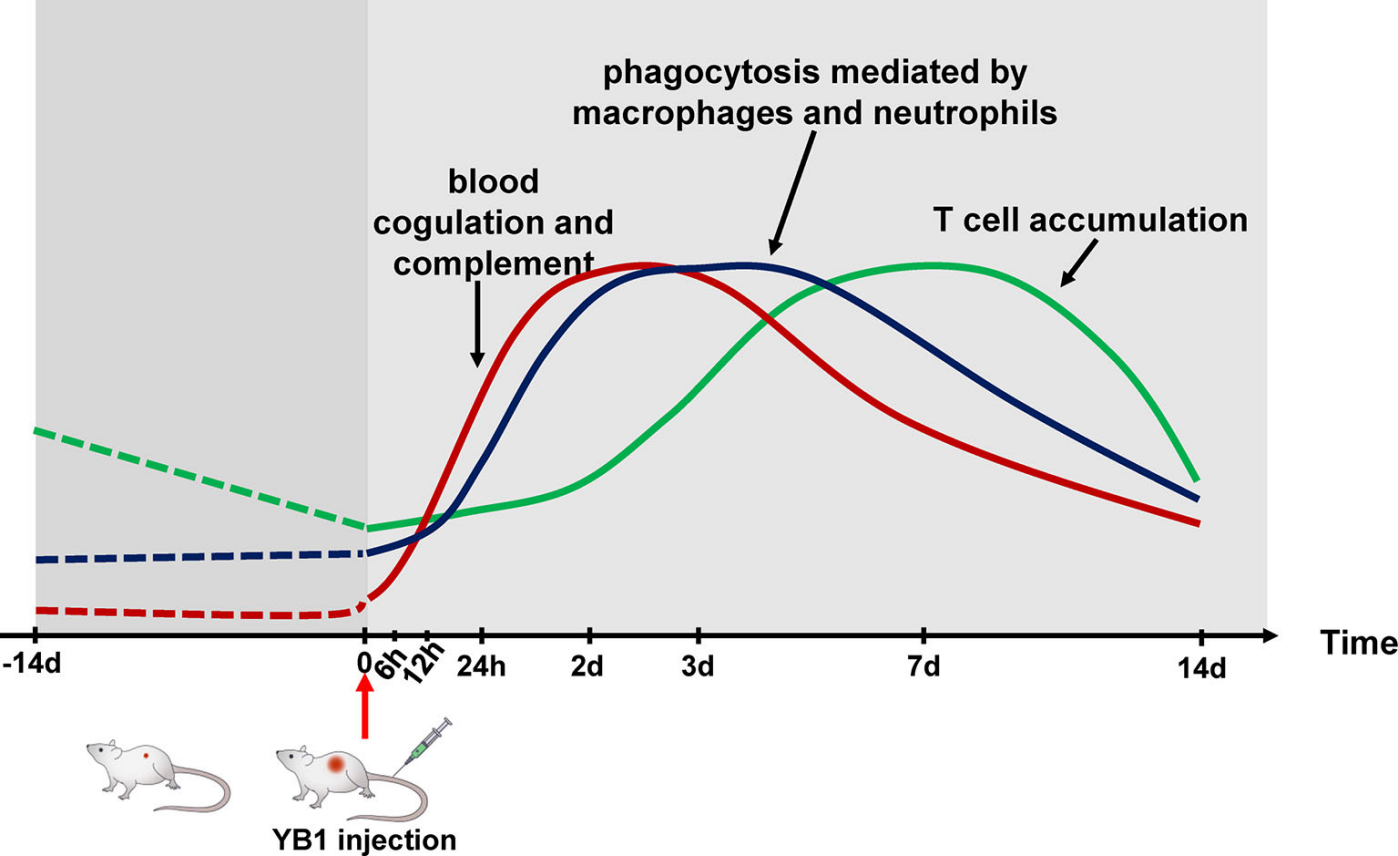
Schematic temporal dynamics of the immunological offensive triggered by YB1 treatment. The red line represents the blood coagulation and complement. The blue line represents phagocytosis mediated by macrophages and neutrophils. The green line represents T cell accumulation in the invasion margin.

The development of a “perfect” bacteria strain for cancer therapy relies greatly on the understanding of host–bacteria interactions (32). Our tumor proteome profiling systematically revealed the dynamics of the tumor proteome after YB1 treatment, which can be beneficial for uncovering the underlying immunological mechanism of YB1-mediated tumor shrink. Nevertheless, this global proteomics approach cannot distinguish proteins from different cell types, therefore, the IHC staining were applied on tumor section images to validate the enrichment of several key marker proteins in the YB1-treated group. Furthermore, the IHC staining can also provide the information on the spatial distribution of various types of cells within tumors. Overall, our present study demonstrated three distinct stages of immune responses induced by YB1 in tumors: the complement and coagulation activation, phagocytosis mediated by macrophage and neutrophils, and T-cell infiltration at the invasion margin of the tumor (**Figure 6**), revealing the complexity of YB1-mediated tumor response at the proteome level.

Our analyses indicate that the complement and blood coagulation pathways were activated soon after YB1 injection. The complement serves as a functional bridge between innate and adaptive immune response that allows an integrated host defense to pathogenic challenges (39). More importantly, angiolysis and thrombosis were observed in the tumors with YB1 treatment. In response to bacteria injection, the innate immune cells and platelets cooperate to induce blood coagulation, which block the nutrition supplement in tumors (33). In addition, YB1 in the tumor anaerobic region could still proliferate robustly and compete with the tumor cells for nutrition (40). It appears that these processes could play important roles in the anti-tumor effects induced by YB1. An independent experiment would be required to validate the phenomena we observed.

Probably due to the presence of the complement component (41) and YB1 aggregation in the anaerobic region, the macrophages and neutrophils are recruited into the tumor to mediate the immune response like phagocytosis. Particularly it is noteworthy that the iNOS^+^ and CD68^+^ cells in the tumors with YB1 treatment was higher than the control group, which may suggest that YB1 could induce anti-tumor effects through improving the infiltration of M1-like macrophages in tumors, though the definition and biological functions of macrophage subpopulations are still under debate (42, 43). It is well known that M1 macrophages play critical roles in innate host defense and killing tumor cells by producing reactive oxygen/nitrogen species (ROS/RNS) and pro-inflammatory cytokines, and hence they are considered as antitumor or “good” macrophages (44, 45). On the other hand, it is worth to note that several inflammatory stimuli can induce the expression of iNOS in various cell types including macrophages, dendritic cells and neutrophils (46–48), the overexpression of iNOS alone does not necessarily indicate the enrichment of M1-like macrophages. Therefore, the reshaping of macrophage subpopulation induced by YB1 remains to be explored further. For the dendritic cells, we did not find the significant difference of the number of CD11c^+^ cells between the YB1 and PBS injection tumors. However, in view of the different subtypes of DCs exhibit differential pro-tumorigenic or anti-tumorigenic functions (49), the contribution of DCs in the bacteria mediated immunotherapy is still to be explored.

As antigen presenting cells, macrophages can present the tumor specific antigens to T cells. In a previous study, the authors demonstrated that the induction of antigen presentation by tumor associated macrophages enhances the accumulation of specific CD8^+^ T cells at the tumor site (50). Furthermore, we found that INF-γ production of spleen CD8^+^ T cells were enhanced after tumor cell challenge, in YB1 treated mice. In addition, the CD3^+^ T cells in the tumors with YB1 treatment accumulated significantly at the invasion margin. As previously described, the presence of T cells at the invasive margin, rather than the center of the tumor, is an important positive predictor of outcome (37, 38). But the mechanism involved in this interplay remains to be investigated.

For collagen in tumors, in our proteomic data, the type I, VI and VII collagens were down regulated. And it was reported that the upregulation of these types of collagen are correlated with the tumor metastasis (51–53). So we hypothesized that the downregulation of the collagen might be involved in the YB1 mediated tumor metastasis inhibition, which could help to explain the reduction of tumor metastasis in YB1 treated tumors (17, 20).

Although bacteria-mediated tumor therapy could be recognized as the oldest type of cancer immunotherapy, its proper clinical application still needs to fulfill very strict requirements of safety and efficacy in both mechanistic and large scale (pre-)clinical studies (8, 54). Early bacteria-based antitumor drug like Coley’s toxin was made of natural pathogen, which showed clear efficacy against multiple types of tumor but suffered from toxicity (4, 8). Nearly Two decades ago, the attenuated salmonella VNP20009 was approved to enter a phase I clinical trial, in which its safety was clearly proved but efficacy was insignificant (55). These days, the emerging synthetic biology technology provided more possibilities for engineering both safe and efficient antitumor bacteria, and combining the synthetic bacteria with other tumor killing agents (13, 15, 54, 56, 57). These advances together shed a light on the future of understanding and applying the bacteria-mediated tumor therapy clinically.

According to our previous studies, YB1 significantly improved the survival rate of tumor-bearing mice in various solid tumor models. On the other hand, in term of safety, YB1 can induce moderate inflammation in the liver and spleen. Nevertheless, within 7 days, the bacteria can be effectively cleared from normal organs (13–17). Furthermore, in a recent study, Lin Q et al. extended the YB1-treatment to several other experimental metastasis models, and found that YB1 has potent suppressive effects on cancer metastasis *via* the host innate immunity (32). Taking all these together, engineered *Salmonella* mediated cancer therapy could be an promising and safe strategy in the future, due to its characteristics including proliferation only in tumors, self-targeting and ease of genetic manipulation (58). Systemic understanding of the curing mechanisms will accelerate its development and application in clinical settings.

In summary, we used a proteomics approach to quantify the temporal changes of the bladder tumor proteome during *Salmonella* YB1 treatment. And the reliability of the proteomics results was verified by the immunohistochemical analysis. We profiled the immunological triple offensive, blood coagulation and complement, phagocytosis, and T-cell infiltration at the tumor invasion margin induced by *Salmonella* YB1, and demonstrate the temporal dynamics of these processes. Our study demonstrated the proteomics technique shows great promises in elucidating mechanisms underlying bacteria mediated immunotherapy.

## Data Availability Statement

The original contributions presented in the study are publicly available. This data can be found here: https://www.ebi.ac.uk/pride/, PXD026288.

## Ethics Statement

The animal study was reviewed and approved by Shenzhen Institutes of Advanced Technology Chinese Academy of Sciences Committee on Animal Care.

## Author Contributions

Conceptualization was carried out by CL, JH, XL, PY, and NL. Investigation was done by SY, WZ, WW, and NL (proteomics), and by SY, MJ, ZZ, JH, and CL (immunohistochemistry, biochemistry and animal experiments), MZ, HH, XL, JB, and PY (data analysis and statistics). The article was written by SY, WZ, MZ, and NL with input from all authors. The figures were made by SY, WZ, MZ, and HH, with input from XL, PY, and NL. The study was supervised by CL, JH, XL, PY and NL. Funding acquisition was carried out by NL and PY. All authors contributed to the article and approved the submitted version.

## Funding

The research was supported by the National Key Research and Development Program of China (2018YFA0902703), the National Natural Science Foundation of China (11801542 31800694 and 31971354), the Natural Science Foundation of Guangdong Province (2020B1515120034), and the Shenzhen Science and Technology Innovation Committee (JCYJ20170818164014753, JCYJ20180507182250795, and JCYJ20180302145723601).

## Conflict of Interest

The authors declare that the research was conducted in the absence of any commercial or financial relationships that could be construed as a potential conflict of interest.

## Publisher’s Note

All claims expressed in this article are solely those of the authors and do not necessarily represent those of their affiliated organizations, or those of the publisher, the editors and the reviewers. Any product that may be evaluated in this article, or claim that may be made by its manufacturer, is not guaranteed or endorsed by the publisher.
