# Supplementary material for: Tumor Temporal Proteome Profiling Reveals the Immunological Triple Offensive Induced by Synthetic Anti-Cancer Salmonella
Source: Front Immunol. 2021 Aug 19;12:712936. doi: 10.3389/fimmu.2021.712936 (PMC8417115; doi:10.3389/fimmu.2021.712936)
Supplement: Supplementary file 1 [file DataSheet_1.pdf]

Supplementary Data

Supplementary table 1 GO terms-biological process at different time points;

Supplementary table 2 List of proteins identified in this study. Dataset 1. Protein expression matrix (56 samples, 4812 proteins)

Supplementary table 3 Dataset 2. A list of proteins (4516 proteins) with at least two unique peptides.

Supplementary table 4 Dataset 3. Protein expression matrix (56 samples, 2739 proteins) identified in at least 2 of the 4 replicates.

Supplementary table 5 List of the differentially expressed proteins in this study (1097 proteins).

Supplementary table 6 Classified modules by k-means clustering of the differentially expressed proteins.

Supplementary table 7 GO annotation of biological processes in the classified modules.

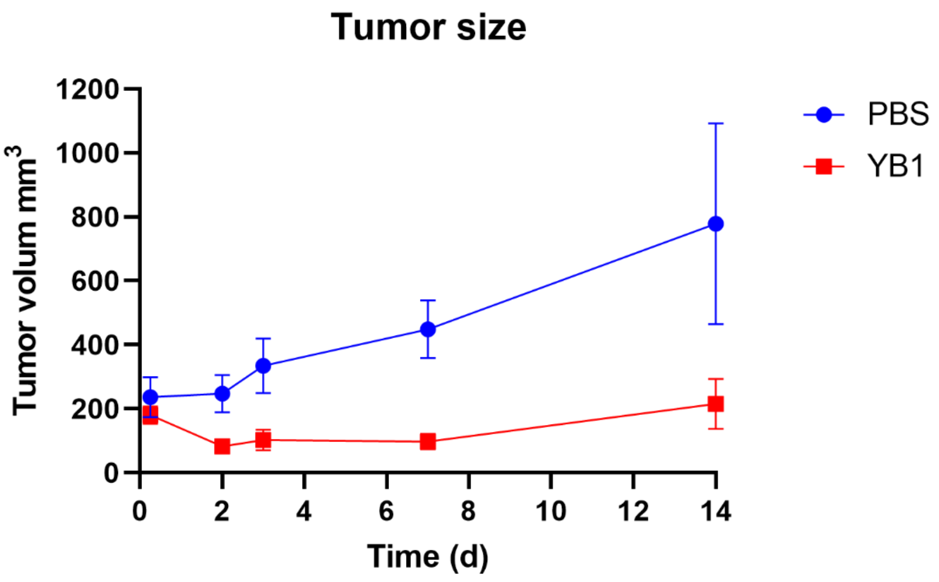

Supplementary Figure 1 Tumor growth curve. The tumor size was measured at 5 time points including 0d, 2d, 3d, 7d and 14d post YB1 treatment. n=4. Statistical analysis was performed using the two-way ANOVA with Šidák's multiple comparisons test. * $p<0.05$, *** $p<0.001$, ****$p<0.0001$

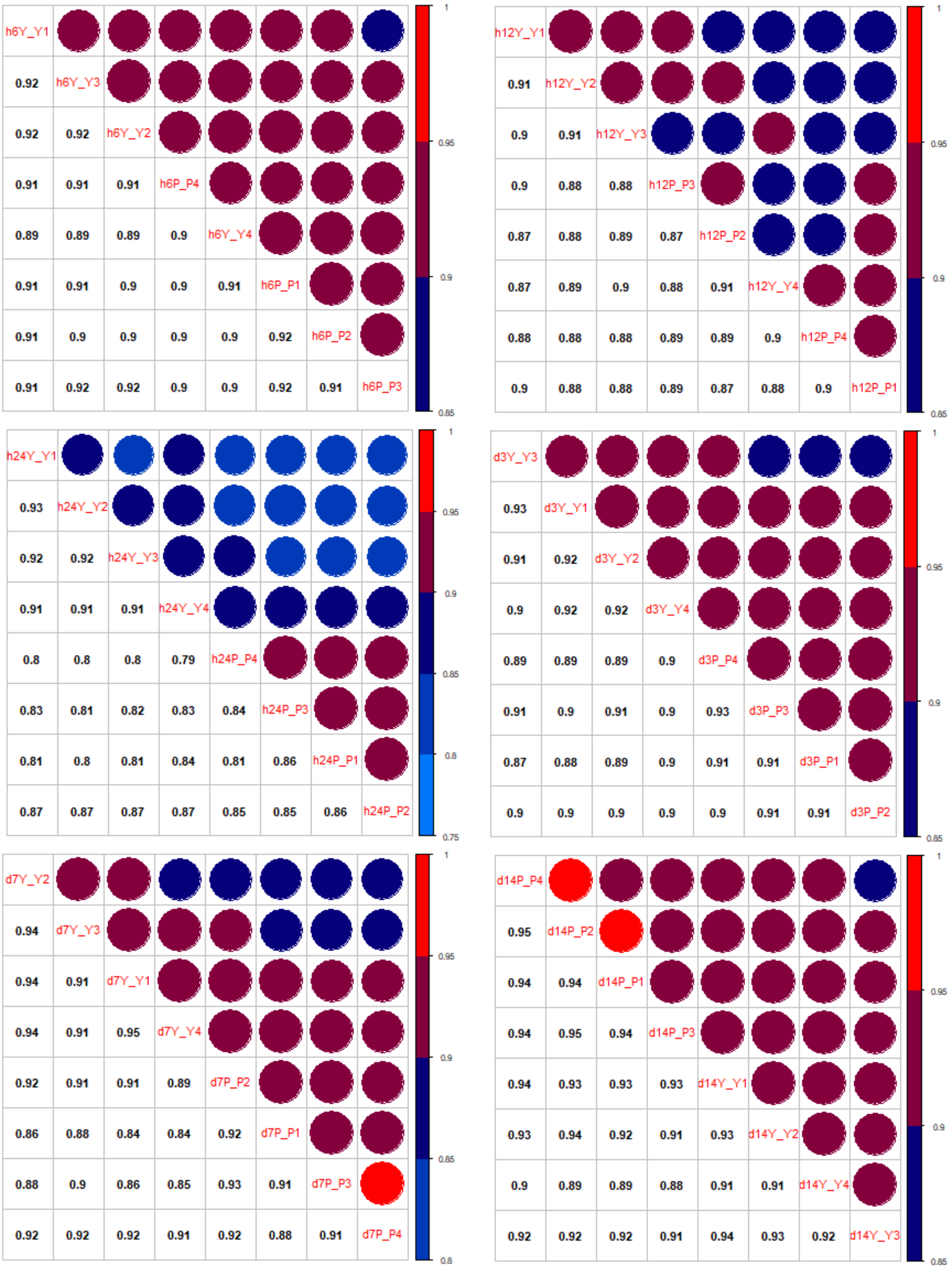

Supplementary Figure 2 Replicate correlation of YB1 *versus* PBS samples at the different time points.

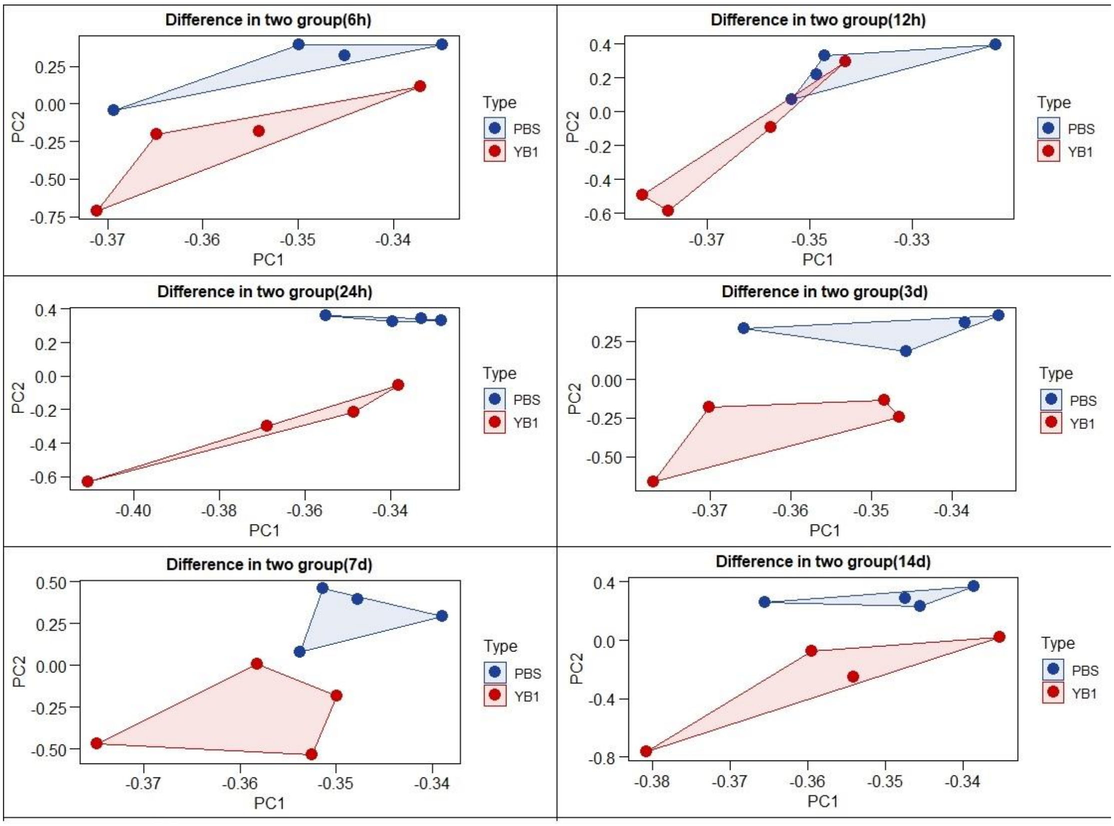

Supplementary Figure 3 Principal component analysis of the proteomics data at different time points. Each dot represents an independent biological replicate.

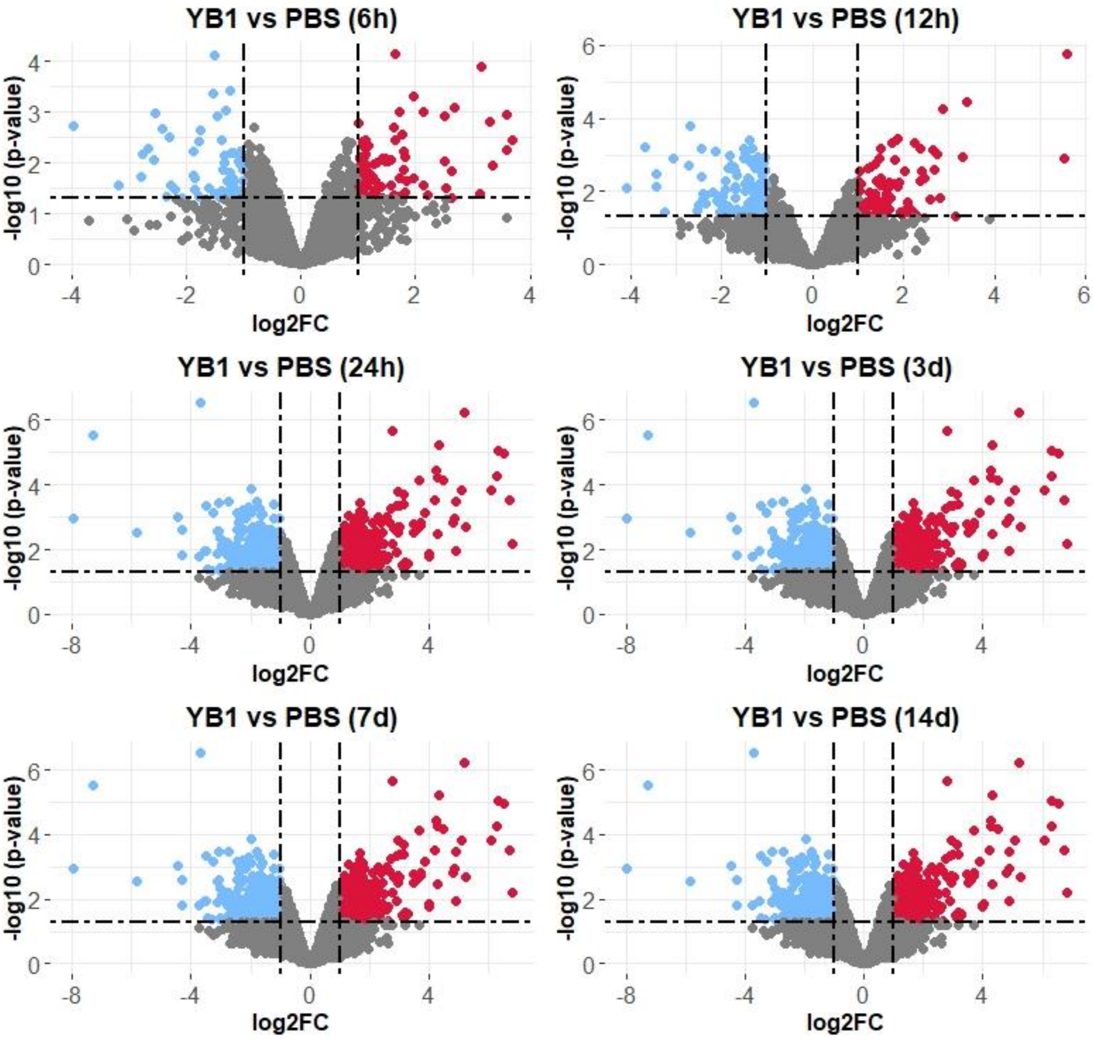

Supplementary Figure 4 Volcano plots illustrating differentially expressed proteins in YB1 versus control samples at different time points. *p*-values from the Limma test (-log base 10) are plotted as a function of the proteins ratio (log base 2) for YB1 *versus* the PBS group. Red dots represent significantly upregulated proteins. Blue dots represent significantly downregulated proteins.
